# Ectopic hyperprolactinaemia due to a malignant uterine tumor resembling ovarian sex cord tumors (UTROCST)

**DOI:** 10.1007/s11102-020-01070-y

**Published:** 2020-08-28

**Authors:** Georgios K. Dimitriadis, Delane S. Wajman, John Bidmead, Salvador J. Diaz-Cano, Sobia Arshad, Mohamed Bakhit, Dylan Lewis, Simon J. B. Aylwin

**Affiliations:** 1 Department of Endocrinology, King’s College Hospital NHS Foundation Trust, Porta Cabin B, Denmark Hill, London, SE5 9RS UK; 2 Division of Translational and Experimental Medicine-Metabolic and Vascular Health, Warwick Medical School, University of Warwick, Coventry, CV4 7AL UK; 3 Department of Urogynaecology, King’s College Hospital NHS Foundation Trust, London, SE5 9RS UK; 4 King’s Health Partners, Cancer Studies, King’s College Hospital NHS Foundation Trust-Viapath, London, SE5 9RS UK; 5 Department of Radiology, King’s College Hospital NHS Foundation Trust, London, SE5 9RS UK

**Keywords:** Hyperprolactinaemia, Ectopic, Paraneoplastic, Extracranial, Prolactinoma, Uterine neoplasm, UTROCST

## Abstract

**Purpose:**

Moderate hyperprolactinaemia (2–5 times upper limit of normal) occurring in a patient with a normal pituitary MRI is generally considered to be due to a lesion below the level of detection of the MRI scanner assuming macroprolactin and stress have been excluded. Most patients with mild-to-moderate hyperprolactinaemia and a normal MRI respond to dopamine agonist therapy. We present the rare case of a patient who had prolactin elevation typical of a prolactin-secreting pituitary macroadenoma,with a normal cranial MRI, and in whom the prolactin rose further with dopamine agonist treatment. Subsequent investigations revealed ectopic hyperprolactinaemia to a uterine tumor resembling ovarian sex cord tumor (UTROSCT) which resolved following tumor resection. Although mostly considered to be benign, the UTROSCT recurred with recurrent hyperprolactinaemia and intraabdominal metastases.

**Methods:**

We have systematically and critically reviewed existing literature relating to ectopic hyperprolactinaemia in general and UTROCST specifically.

**Results:**

Fewer than 80 cases of UTROSCTs have been reported globally of which about 23% have shown malignant behaviour. There are fewer than 10 cases of paraneoplastic hyperprolactinaemia originating from uterine neoplasms including one other case of ectopic hyperprolactinaemia to a UTROSCT.

**Conclusions:**

Our case demonstrates the importance of screening for extracranial hyperprolactinaemia in the context of: (1) substantially raised prolactin (10× ULN) and (2) normal cranial MRI assuming macroprolactin has been excluded. The majority of extracranial ectopic prolactin-secreting tumors occur in the reproductive organs.

## Introduction

The human prolactin (PRL) gene, located on chromosome 6 [1], apparently rose from a single common ancestral gene giving rise to the relatively homologous PRL, growth hormone (GH), and the placental lactogen-related proteins [2]. Several factors influence PRL, gene expression, including oestrogen, dopamine, thyrotropin-releasing hormone (TRH), and thyroid hormones [3]. PRL secretion is under the inhibitory control of dopamine, which is produced mainly by the tuberoinfundibular cells and the hypothalamic tuberohypophyseal dopaminergic system [4, 5]. High concentrations of circulating prolactin are typically associated with either prolactin secreting pituitary tumors or interference with the dopaminergic control of prolactin from normal pituitary such as pituitary stalk disconnection and dopamine agonist medications, as well as other conditions including primary hypothyroidism, uraemia and hepatic insufficiency.

Apparently high levels of prolactin may be due to excess circulating prolactin complexes (‘macroprolactin’) which need to be differentiated from true hyperprolactinaemia. Increased prolactin may also rise due to acute medical stress and therefore diagnostic levels of prolactin should be ideally taken after intravenous cannulation [6].

The ectopic production of hormones from tissues that are not generally considered to be part of their usual cellular behaviour are a consistent theme in endocrinology and lead to distinct clinical syndromes. These syndromes can originate from either endocrine or non-endocrine neoplasms. They can precede, occur concomitantly, or present at a later stage of neoplasm development, and along with the secreted substances constitute the biological ‘fingerprint’ of the neoplasm. Their detection can facilitate early diagnosis of the underlying neoplasia, monitor response to treatment, and/or detect early recurrences following successful initial management [7].

Ectopic prolactin may arise from prolactinoma cells that have arisen intracranially or within the sinuses and these likely arise from normal pituitary cells that are remnants left during the development of the pituitary from Rathke’s pouch. These have the cellular appearance of typical prolactinoma but are “extrapituitary”. There are in addition a very small number of cases where prolactin has been released from tissues that do not usually express prolactin and are most frequently associated with neoplasms of the female reproductive system. In this report we have identified hyperprolactinaemia arising due to ectopic production from a malignant uterine tumor resembling ovarian sex cord tumor (UTROSCT) [8].

## Case presentation

**Fig. 1:**
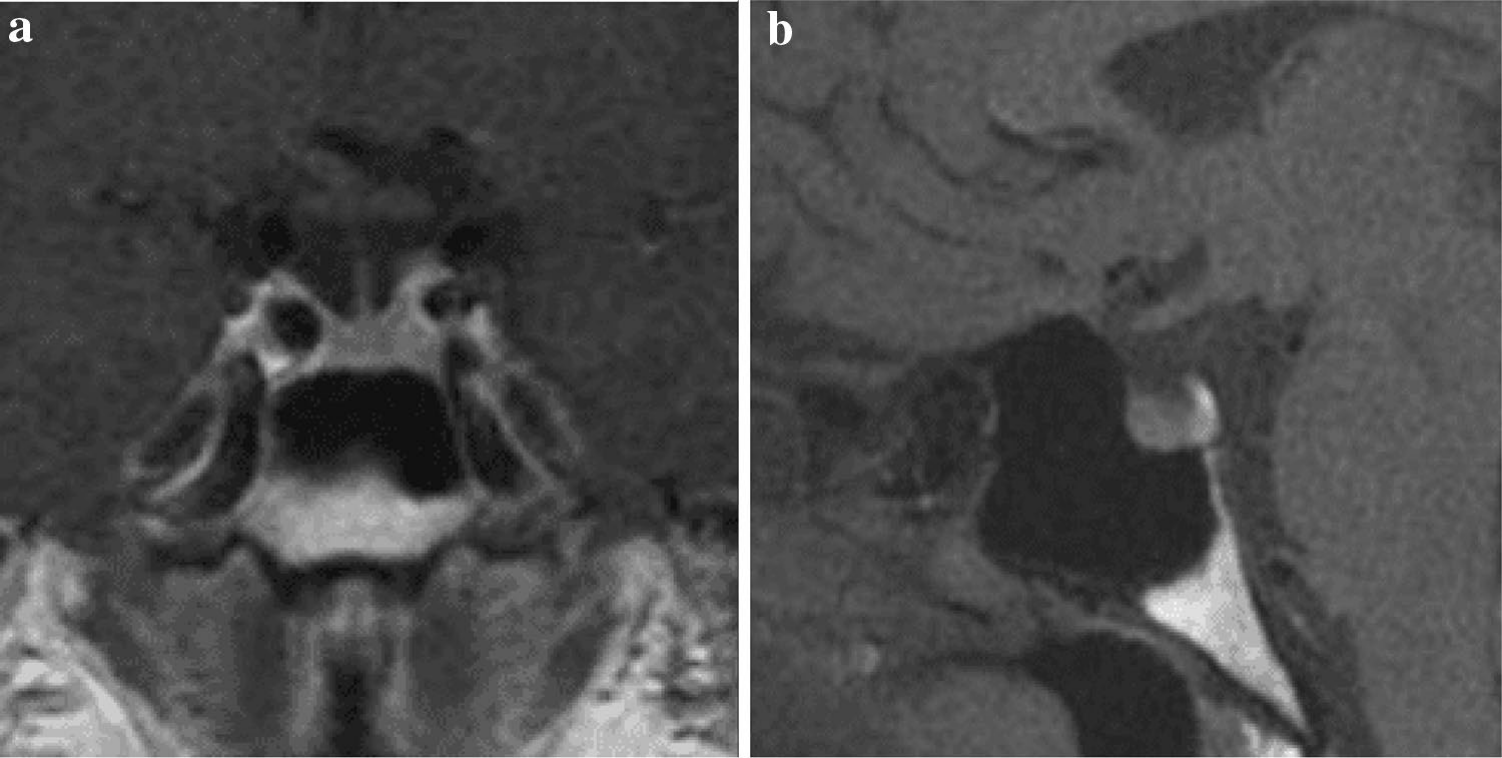
Targeted MRI examination of the pituitary region including coronal and sagittal T1-weighted sequence post-contrast enhanced images revealing no abnormalities

A 46-year-old Caucasian female presented to the endocrine outpatient clinic with a 12 month history of secondary amenorrhoea. She was a mother of two children following spontaneous conception and previously used a progestin releasing intrauterine device which had been removed 6 months before amenorrhoea was first observed. She was not on any regular medications. On initial biochemistry tests, prolactin was 223 mcg/L (reference range 4–23 mcg/L for premenopausal non-pregnant women) without macroprolactin complexes following a precipitation test. LH & FSH were low (0.1 and 1.5 mIU/L respectively with reference range 2–12 mIU/L during follicular phase) and oestradiol was undetectable (< 55 nmol/L, reference range > 100 nmol/L for premenopause), with normal thyroid function tests (TSH: 1.04 mU/L; reference range 0.30–5.50 mU/L and fT4: 13.4 pmol/L; reference range 9–26 pmol/L). She had normal visual fields on confrontation and no other clinical or biochemical features of pituitary dysfunction. A subsequent contrast-enhanced pituitary magnetic resonance imaging (MRI) scan showed normal pituitary gland appearances without evidence of a focal lesion (Fig. 1a, b).

She was started on cabergoline 250 mcg twice weekly, which was subsequently increased to 500 mcg twice weekly. Repeat serum prolactin 5 months and 8 months later showed a progressive rise to 313 mcg/L and 454 mcg/L respectively, rising further to 546 mcg/L despite further intensification of dopamine agonist treatment. Compliance with medication was confirmed and a repeat pituitary MRI scan was again normal.

**Fig. 2:**
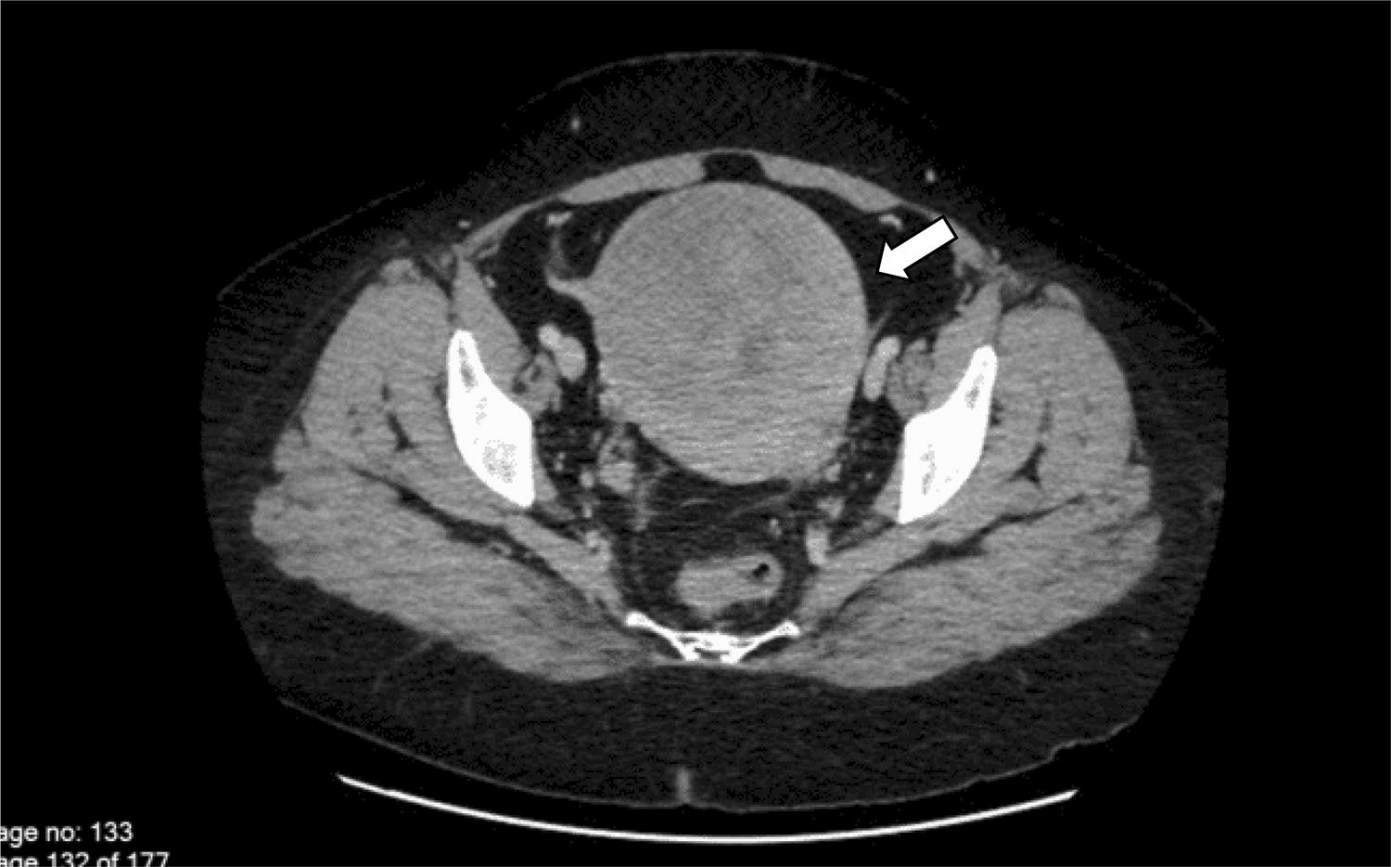
Pelvic CT examination revealing a well-defined 11 cm pelvic mass without further abnormalities

**Fig. 3:**
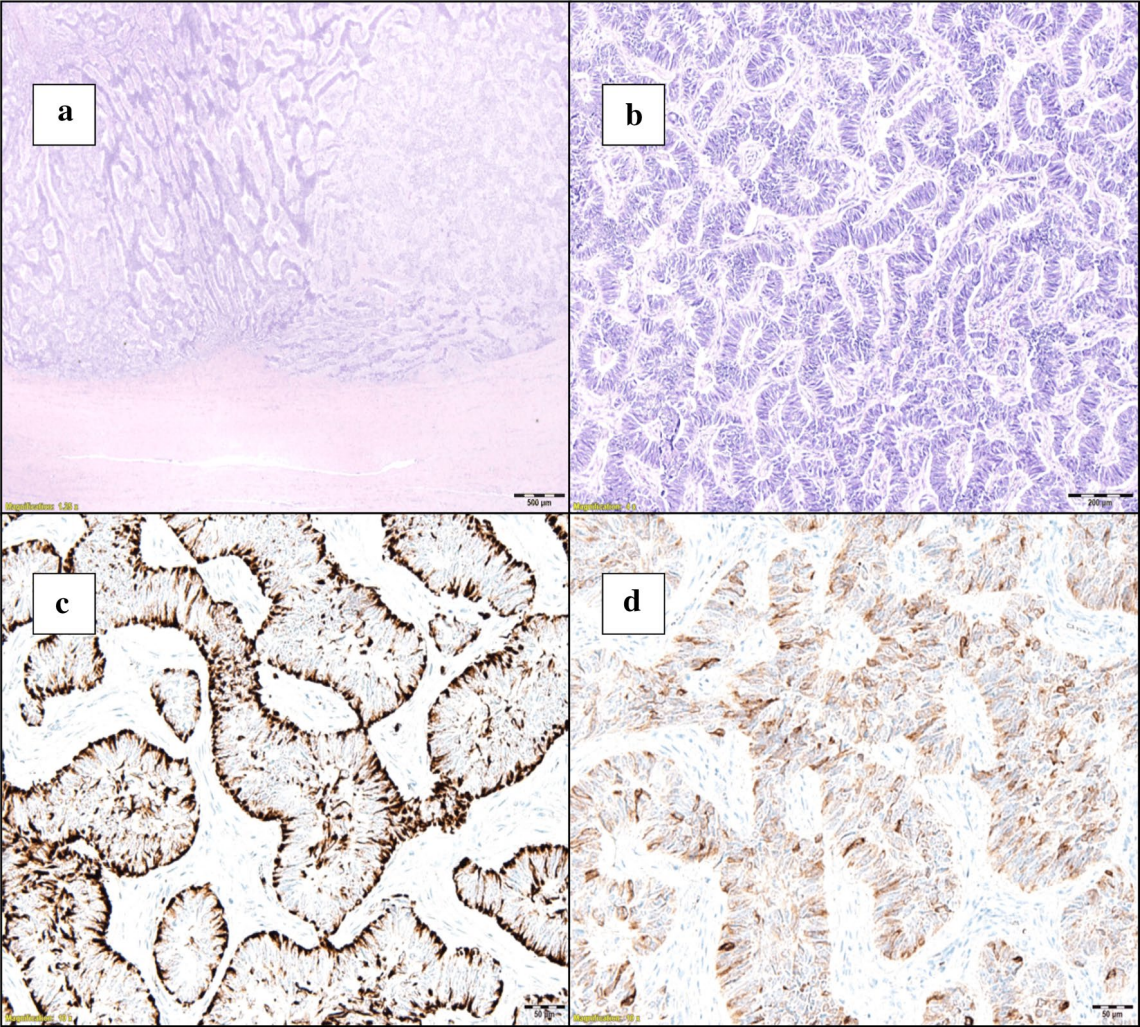
Immunohistochemical staining and molecular analyses. Immunostaining in cord-like and solid elements: endometrial glands are infiltrated by WT1-positive tumor cells; magnification ×1.25 (**a**), CD10 shows variable positivity in areas; magnification ×1 (**b**). Immunostaining in cord-like and solid elements: α-smooth muscle actin is focally positive in the solid lesion; magnification ×10 (**c**), whereas cytokeratin (AE1/AE3) is expressed diffusely in the cord-like area; magnification ×10 (**d**)

**Fig. 4:**
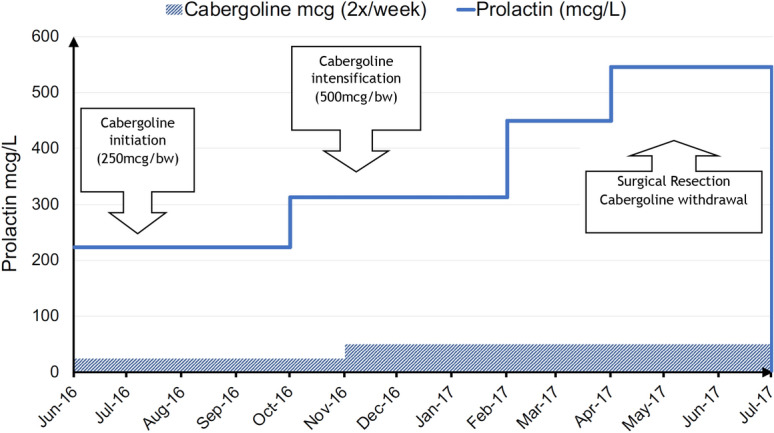
Prolactin values in mcg/L (reference rage 4–23 mcg/L for premenopausal non-pregnant women) during different disease stages, illustrating resistance and biochemical disease progression on DA therapy with complete biochemical remission following surgery

At this stage an alternative source of prolactin secretion was considered and since ectopic prolactin has been recognised as being a feature of neoplasms of the reproductive organs, further clinical assessment revealed a palpable pelvic mass in the lower abdomen rising to the umbilicus. Pelvic computerised tomography (CT) scan showed an 11 cm uterine mass initially reported as a fibroid which raised the suspicion of an ectopic source of hyperprolactinaemia (Fig. 2). She underwent total hysterectomy and histological examination showed a well-circumscribed but encapsulated UTROSCT in which cells were organized in sheets, cords, nests, and trabeculae. Ki-67 was < 5%, and mitotic count was 6 per 10HPF (Fig. 3). Immunohistochemistry (IHC) was negative for prolactin, melanA, and Calretinin. It was positive for inhibin in the trabecular areas, CD99 was focally positive, desmin was positive, CD10 focally weakly positive, ER, PR, AE1/AE3 and SMA were focally positive (Table 1). Despite negative tissue staining for prolactin, postoperatively serum prolactin concentration was entirely suppressed at 3 mcg/L (Fig. 4). In view of the finding of a neoplasm with malignant potential, further surgery was recommended and she underwent bilateral salpingo-oophorectomy. After 2 years however, the patient was found to have evidence of intraabdominal recurrent tumor and was then proven again to have elevated prolactin.

**Table 1:** Source and condition of antibodies, and summary of immunohistochemistry

Antibody	Source	Working dilution	IHC result
CK (AE1/AE3)	Progen	1:600	++
CD10	Roche	Prediluted	+ (focally)
Desmin	Nichirei	Prediluted	++
αSMA	Nichirei	Prediluted	+ (focally)
Calretinin	Nichirei	1:50	−
CD56	Nichirei	Prediluted	+ (focally)
WT1	Nichirei	Prediluted	+ (focally)
CD99 (MIC2)	DAKO	1:100	+ (focally)
ER	Roche	Prediluted	+ (focally)
PgR	Roche	Prediluted	+ (focally)
PRL	Roche	Prediluted	−
Inhibin	DAKO	1:25	++
Melan A	Novo	1:20

## Methods

We conducted systematic searches of the Medical Literature Analysis and Retrieval System Online (MEDLINE) and Excerpta Medica (EMBASE). The Medical Subject Headings (MeSH) search terms included ectopic, or paraneoplastic, and hyperprolactinaemia, or hyperprolactinemia and the search strategy for MEDLINE was constructed as follows: *((((ectopic) OR paraneoplastic) AND hyperprolactinaemia) OR hyperprolactinemia).* Our systematic search revealed a total of 132 reports in the literature, most of which of cranial origin, including ectopic pituitary adenomas [9–11]. Our search identified six other reports of hyperprolactiaemia to a uterine neoplasm with only one case reporting this being secondary to a UTROSCT. These reports are summarised in Table 2.

**Table 2:** Summary of cases reporting on hyperprolactinaemia to a uterine neoplasm

Case	Year	Age (years)	Presenting symptoms	PRL concentration (mcg/L)	Response to medical treatment	Location of tumour	Tumour size (cm)	Surgical treatment	PRL IHC— Histology
Coridano V.	2005	44	Amenorrhoea Erythrocytosis	218	No response	Uterus	8	Hysterectomy	Not performed Leiomyoma
Herzog AG	2000	36	Amenorrhoea Galactorrhoea Migraines	100	Deterioration	Uterus	5.5	Hysterectomy	Not performed Leiomyoma
O’Meara AC	2009	35	Galactorrhoea Metrorrhagia	87	None received	Uterus	8.6	Hysterectomy BSO	Negative— UTROSCT
Sato et al.	2017	45	Amenorrhoea	91.5	No response	Uterus	9	Hysterectomy	Negative—Leiomyoma
Sendur et al.	2019	25	Amenorrhoea Galactorrhoea Subfertility	150	No response	Uterus	8	Myomectomy	Negative—Leiomyoma
Simsir IY et al.	2012	34	Amenorrhoea Galactorrhoea	152	Deterioration	Uterus	15	Hysterectomy	Negative Mesenchymal tumour
Our case	2020	46	Amenorrhoea	223	Deterioration	Uterus	11	Hysterectomy	Negative —UTROSCT

*PRL* prolactin, *IHC* immunohistochemistry, *UTROSCT* uterine tumours resembling ovarian stress cord tumours

## Discussion

Hyperprolactinaemia is a common presenting problem in endocrine practice. It is more common in women where the incidence is 0.2% in the general adult population and 17% in women with a reproductive disorder [12]. Once stress-related hyperprolactinaemia and macroprolactin are excluded, the most frequent cause is a prolactin secreting pituitary adenoma [13, 14].

An alternative aetiology is that of idiopathic hyperprolactinaemia, responsible for 3.6% of all cases [13, 14]. It is defined as clinical or asymptomatic hyperprolactinaemia in the absence of macroprolactin with negative pituitary imaging after pathological causes such as hypothyroidism, renal, and severe liver insufficiency as well as other hyperprolactinaemia causes have been previously excluded [14]. Small microprolactinomas less than 3 mm in max diameter, below the threshold of detection on MRI, are the likely cause [14]. An alternative cause of the “idiopathic hyperprolactinemia” phenomenon may be the presence of anti pituitary antibodies [15]. While the normal endometrium retains the ability to discharge PRL in the late luteal phase. Studies on PRL levels in the peritoneal fluid show no evidence that the implants secrete prolactin [16].

“Ectopic” or paraneoplastic secretion of a hormone refers to the production by a cell type that does not typically produce that substance or produces it at low concentrations. Ectopic hormone production may also by a hormone-producing cell whose “machinery” has been co-opted to produce another hormone. An example of this is the production of adrenocorticotrophic hormone (ACTH) by a broad spectrum of tumor types with some degree of neuroendocrine differentiation, such as small cell lung carcinomas (SCLCs) [7].

In this report we describe a patient whose presentation was due to hyperprolactinaemia due to a UTROSCT which initially resoved after tumor resection but then demonstrated malignant behaviour.

Ectopic hyperprolactinaemia is rare and can be considered intracranial or extracranial. Ectopic intracranial production of prolactin arises from tumors that have typical features of pituitary prolactinomas and are thought to originate from the neoplastic proliferation of pituitary rests along the embryological path of the pituitary development [17, 18]. Approximately 60% of reported ectopic pituitary adenomas are seen in the sphenoid sinus and suprasellar region, and 30% can be found in the clivus, nasal cavity, cavernous sinus, parasellar region, and sphenoid wing [19]. Rare sites beyond the migrational tract have also been described, including those in the petrosal temporal bone [20], superior orbital fissure [21], third ventricle [22], and temporal lobe [23]. These tumors typically respond to dopamine agonist to the same extent as eutopic prolactinomas.

Extracranial hyperprolactinaemia is even rarer and has previously been reported in both benign and malignant neoplasms [24, 25]. Elms et al. recently reported a case of significant hyperprolactinaemia from a benign cystic teratoma containing a prolactin-secreting pituitary adenoma with resolution following resection of the tumor [26]. Kallenberg et al*.* and Gururaj et al*.* also reported hyperprolactinaemia to an ovarian teratoma [27, 28]. Hyperprolactinaemia from uterine dermoid tumors has been reported by others previously [26]. Dermoid tumors can differentiate into other types of cells acquiring their secretory components, thus making them a potential source of ectopic hyperprolactinaemia [29, 30]. In the limited previous reports of ectopic extracranial prolactin secretion in the published literature, ovarian germ cell tumors associated with hyperprolactinaemia had microscopic pituitary elements [25–28]. Turkington et al*.* have reported two cases of ectopic prolactin production. In one case, from an undifferentiated bronchial carcinoma and another case of hyperprolactinaemia from a hypernephroma [22].

There have been fewer than 10 cases where excess prolactin has been attributed to a uterine neoplasm as summarised in Table 2. Interestingly, in all these cases the tumors failed to stain for prolactin despite the hyperprolactinaemia resolving after surgery. Cordiano reported the case of hyperprolactinaemia to a uterine leiomyoma in a patient with a co-existent pituitary microadenoma not responding to treatment with bromocriptine [31]. Herzog also reported hyperprolactinaemia to a uterine leiomyoma presenting with migraine and no response to treatment with bromocriptine [32]. Symptoms resolved following a hysterectomy. Sato et al*.*, report the case of a Japanese female patient with refractory hyperprolactinaemia and negative MRI for pituitary neoplasm with symptom resolution following hysterectomy for a leiomyoma [33]. Sendura et al*.* report the case of a patient with persistent hyperprolactinaemia, secondary amenorrhoea, and galactorrhoea despite being treated with bromocriptine and cabergoline previously. Hyperprolactinaemia was secondary to a giant leiomyoma with negative immunohistochemistry for PRL [34]. Simsir et al*.*, report the case of hyperprolactinaemia with associated secondary amenorrhoea and galactorrhoea to a leiomyosarcoma with complete resolution of symptoms following hysterectomy. Immunohistochemistry was negative for PRL [35]. O’meara et al*.* report the only other case of hyperprolactinaemia with abdominal discomfort, galactorrhea and metrorrhagia to a UTROSCT with complete resolution of symptoms following total abdominal hysterectomy. Immunohistochemistry was negative for PRL [36]. Under certain conditions, specific alterations of gene function may inappropriately herald the unscheduled appearance of a gene product at an unusual time of life or in an atypical cell, tissue or organ leading to a paraneopastic syndrome. However, even though cells acquire this ability under gene manipulation, they cannot alter their secretory features and may not be able to store synthesised peptides leading to negative staining [7].

Patients with UTROSCT typically present with metrorrhagia and/or a uterine mass [8, 36]. This category of uterine neoplasms with variable biological behaviour is divided into two groups: uterine tumors resembling ovarian sex cord tumors (UTROSCT), and the related endometrial stromal tumors with sex cord-like elements (ESTSCLE) [37]. According to World Health Organisation (WHO), UTROSCT is currently defined as the “neoplasm resembling ovarian sex cord tumors without a component of recognizable endometrial stroma” (WHO, I.A.R.C. 2014, 4th Ed) [38]. UTROSCTs are rare uterine neoplasms with literature citing less than 80 cases in total, with only one case associated with ectopic prolactin. However, other reported cases of ectopic hyperprolactinaemia with uterine tumors have common characteristics with this case and it is possible that these may have potentially belonged to the same tumor category [25].

The cardinal features of our case were: the high prolactin suggestive of a macroadenoma, a normal MRI scan, a paradoxical rise in serum prolactin despite initiation of dopamine agonist therapy, and resolution of hyperprolactinaemia after surgical excision. Although hyperprolactinaemia of extracranial origin is exceptionally rare, it should be suspected in patients found to have very high (> 10× ULN) serum prolactin concentrations and normal findings on cranial imaging. In cases of ectopic hyperprolactinaemia in premenopausal female patients this would be most often demonstrated by imaging of the reproductive system.
